# fNIRS Hemodynamic Alterations in Developmental and Acquired Language Disorders and Dyslexia: An Exploratory Transdiagnostic Systematic Review and Meta-Analysis

**DOI:** 10.3390/diagnostics16152388

**Published:** 2026-07-29

**Authors:** Qi An, Chuanyu Yang, Yuyuan Zeng, Jianhong Wang, Bo Zhou, Wenquan Niu, Yike Zhu, Mengjiao Tao, Lin Wang

**Affiliations:** 1 Capital Center for Children’s Health, Capital Medical University, Capital Institute of Pediatrics, Beijing 100020, China; anqi20190220@163.com (Q.A.); yangchuanyu1234@163.com (C.Y.); 13365065979@163.com (Y.Z.); child811117@163.com (J.W.); bobo-0207@hotmail.com (B.Z.); a349841551@163.com (Y.Z.); taomengjiao@foxmail.com (M.T.); 2 Capital Institute of Pediatrics, Chinese Academy of Medical Sciences & Peking Union Medical College, Beijing 100020, China; 3 Center for Evidence-Based Medicine, Capital Center for Children’s Health, Capital Medical University, Beijing 100020, China; niuwenquan_shcn@163.com

**Keywords:** functional near-infrared spectroscopy, language disorders, dyslexia, hemodynamic responses, systematic review, meta-analysis

## Abstract

**Background**: Language disorders and dyslexia are associated with atypical language- and reading-related processing, but findings from functional near-infrared spectroscopy (fNIRS) studies remain heterogeneous. This systematic review and meta-analysis synthesized existing fNIRS evidence, quantified hemodynamic differences relative to controls, and examined whether different outcomes showed different levels of consistency. **Methods**: PubMed, Embase, and Web of Science were searched through 9 January 2026. Eligible studies included individuals with language disorders or dyslexia assessed using fNIRS, with outcomes reported as oxygenated hemoglobin (HbO), deoxygenated hemoglobin (HbR), β estimates, or other hemoglobin-based measures. Random-effects meta-analyses were performed when sufficient quantitative data were available, with subgroup and sensitivity analyses conducted where feasible. **Results**: A total of 830 records were identified, of which 10 publications comprising 11 independent samples met the inclusion criteria. HbO was the only outcome to show a statistically significant overall between-group difference, with lower HbO in the experimental groups than in controls (SMD = −0.61, 95% CI [−1.12, −0.11], *p* = 0.017), although heterogeneity was substantial (I^2^ = 58.6%). In contrast, pooled results for HbR and β were not statistically significant and showed weaker stability across analyses. Subgroup and sensitivity analyses did not provide sufficient evidence to support disorder category, region of interest (ROI), or outcome metric type as stable explanations for effect size variation. **Conclusions**: Among the included fNIRS measures, HbO showed comparatively more consistent cross-study evidence than HbR or β. However, because the HbO estimate pooled clinically distinct dyslexia and acquired language-disorder studies within a limited evidence base, it should be interpreted as a preliminary cross-diagnostic signal rather than as evidence of a shared disease continuum or common underlying mechanism.

## 1. Introduction

Language and reading-related disorders can occur at various stages from childhood to adulthood, significantly impacting an individual’s communication, learning, and social functioning.

Language disorders can arise from a variety of causes. According to the CATALISE consensus, language disorder refers to difficulties with language that interfere with everyday communication or learning and are likely to persist over time [1]. In the present review, this category includes both developmental and acquired conditions. Developmental language disorder (DLD) is typically characterized by deficits in language comprehension and expression. Its neural basis is considered to exhibit networked characteristics [2]. However, acquired language disorders, such as post-stroke aphasia, result from damage to previously developed language systems [3]. Although developmental and acquired language disorders differ in onset and etiology, both involve abnormalities in language-processing networks and may therefore provide insight into altered language-related hemodynamic responses.

Dyslexia is generally characterized by significant difficulties in word-level reading and decoding, particularly in establishing and automatizing letter–sound mappings, which cannot be fully explained by general intellectual impairment or inadequate educational opportunity [4,5]. Neuroimaging studies have further shown that dyslexia is associated with atypical patterns of functional activation and connectivity in neural systems supporting language and reading.

From a neurobiological perspective, language disorders and dyslexia are not identical, but they are comparable in that both involve atypical functioning within language-related neural networks, particularly frontotemporal systems. Their differences may lie in the relative contribution of specific network components and in the functional activation patterns associated with different processing demands [2,3,6]. Therefore, the inclusion of both conditions in a single meta-analysis does not assume shared underlying mechanisms. Rather, it reflects the transdiagnostic question addressed in the present review: whether abnormal hemodynamic responses measured during language-related tasks show sufficiently comparable patterns across language disorders and dyslexia to support synthesis within a unified analytical framework.

In the study of language disorders and dyslexia, reliance on behavioral measures alone is often insufficient to fully elucidate their underlying mechanisms. Therefore, neuroimaging techniques are needed to further investigate the atypical processing mechanisms underlying these two disorders at the level of brain function. However, not every neuroimaging technique is suitable for language-related research. For instance, functional magnetic resonance imaging (fMRI), despite its high spatial resolution, is highly sensitive to head movement, exhibits significant environmental noise during scanning, and requires high subject compliance [7]. Electrophysiological techniques such as EEG offer excellent temporal resolution, but their ability to localize cortical activity is relatively limited [8]. Additionally, in natural tasks involving reading and speech production, electromyographic (EMG) artifacts induced by articulation may mask or interfere with neural signals, thereby increasing uncertainty in result interpretation [9].

Functional near-infrared spectroscopy (fNIRS), a non-invasive, highly portable, and minimally restrictive brain imaging technique, offers new possibilities to address certain limitations of conventional methods in developmental populations and natural language task research. It enables real-time monitoring of hemodynamic changes in cortical activity during specific task execution with high temporal resolution [10].

It is based on the principle of neurovascular coupling and indirectly reflects local cerebral hemodynamic responses to neural activity by measuring temporal changes in the concentrations of oxyhemoglobin (HbO) and deoxyhemoglobin (HbR) in cortical regions. HbO typically exhibits higher sensitivity to task-related activation and is therefore widely used as the primary analytical indicator in fNIRS studies. HbR, on the other hand, is often employed as a supplementary indicator to provide further characterization of hemodynamic change patterns [11]. In addition, some studies have adopted β coefficients derived from general linear model (GLM) analyses as outcome measures to quantify the magnitude of hemodynamic responses elicited by specific task conditions or contrasts. By analyzing the variation patterns of HbO, HbR, and β under different task conditions, researchers can infer the functional involvement of cortical regions and their temporal dynamic characteristics during specific cognitive or language processing tasks.

In language and reading research, fNIRS-derived hemodynamic measures have increasingly been used to examine the functional characteristics of cortical regions involved in sentence, semantic, and reading processes [12,13]. In individuals with language disorders and dyslexia, previous studies have reported atypical hemodynamic responses during such tasks relative to typical controls, suggesting differences in the organization of cortical activation. Therefore, fNIRS studies based on HbO, HbR, and model-derived activation measures provide valuable hemodynamic evidence relevant to understanding the neural mechanisms underlying language disorders and dyslexia.

Although systematic reviews have summarized the application of fNIRS in speech and language disorders, studies that simultaneously examine language disorders and dyslexia and quantitatively synthesize hemodynamic outcomes remain scarce. Existing reviews have focused primarily on participant characteristics, task paradigms, and study design, whereas quantitative integration of HbO, HbR, and related functional activation measures has been limited [7,14]. Moreover, substantial heterogeneity in outcome definitions and reporting practices across studies has hindered direct comparison and cumulative interpretation of findings. Therefore, a systematic synthesis within a unified framework is needed to quantify abnormal hemodynamic response patterns in language disorders and dyslexia and to provide preliminary clues for potential commonalities and specificities among different types of disabilities.

Specifically, this review sought to address the following questions:

(1) Do individuals with language disorders and dyslexia show altered hemodynamic responses during related tasks relative to healthy controls?
(2) Do these hemodynamic differences vary according to regions of interest and other study characteristics?
(3) Do different outcome metrics and analytical frameworks, such as HbO, HbR, and model-derived β estimates, show different levels of stability and comparability in characterizing these differences?
(4) Do language disorders and reading disorders show a degree of directional similarity or divergence in their hemodynamic response patterns, thereby providing exploratory evidence for transdiagnostic features and disorder-specific differences?

## 2. Materials and Methods

### 2.1. Search Strategy

This systematic review and meta-analysis was conducted and reported in accordance with the PRISMA 2020 statement [15]. A comprehensive literature search of PubMed, Embase, and Web of Science was conducted from database inception to 9 January 2026. The search strategy was structured around two main concepts: (1) functional near-infrared spectroscopy and related terms, including “functional near-infrared spectroscopy,” “near-infrared spectroscopy,” “near-infrared spectrometry,” “fNIRS,” and “NIRS”; and (2) language- and reading-related disorders, including “language disorders,” “developmental language disorder,” “specific language impairment,” “aphasia,” “dyslexia,” “alexia,” “reading disorder,” and “reading disability.” Searches were conducted in title/abstract fields in PubMed, title/abstract/keyword fields in Embase, and Topic fields in Web of Science. English-language publication was applied as an eligibility criterion. Only studies published in English were considered. The full search strategies for all databases are provided in Table S1. The review protocol was registered in PROSPERO on 24 February 2025 (registration number: CRD420250652628).

### 2.2. Eligibility Criteria

Studies were considered eligible if they met the following inclusion criteria: (1) Study types: Observational studies; (2) Participants: Individuals diagnosed with language disorders or dyslexia; (3) Detection tool: Functional near-infrared spectroscopy (fNIRS); (4) Comparator: Typically developing individuals, healthy controls, or other relevant comparison groups; (5) Outcome: fNIRS-derived hemodynamic measures, including HbO, HbR, and β estimates, with other hemoglobin-based measures extracted where relevant for narrative synthesis.

Studies were excluded if they fulfilled the following exclusion criteria: (1) Studies primarily designed to evaluate treatment or intervention effects were excluded, unless eligible pre-intervention baseline data were available; (2) case reports, conference abstracts, letters, comments, editorials, review articles, and non-English publications; (3) studies not involving fNIRS; (4) studies without eligible language disorder or dyslexia groups.

### 2.3. Study Selection Process

Title and abstract screening was conducted independently by two reviewers based on the predefined eligibility criteria. Articles considered potentially relevant were then retrieved in full and assessed for inclusion. Disagreements were resolved through discussion, and when consensus could not be reached, a third senior reviewer was consulted.

**Figure 1:**
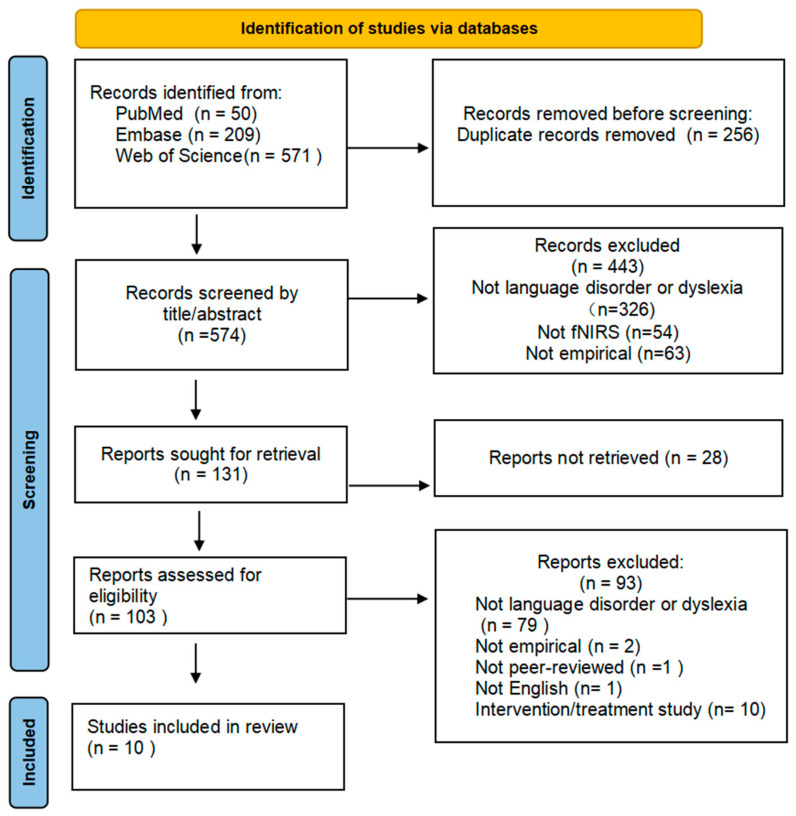
PRISMA flow diagram showing the identification, screening, eligibility assessment, and inclusion process.

Following the literature search, 830 records were identified from PubMed, Embase, and Web of Science. After removal of 256 duplicate records, 574 records were screened by title and abstract. At this stage, 443 records were excluded because they were not related to language disorders or dyslexia, did not involve fNIRS, or were not empirical studies. The remaining 131 reports were sought for full-text retrieval, of which 28 could not be retrieved. A total of 103 full-text reports were then assessed for eligibility. Of these, 93 reports were excluded because they did not meet the population criteria, were not empirical or peer-reviewed studies, were not published in English, or were intervention/treatment studies without eligible baseline data. Finally, 10 studies met the inclusion criteria and were included in the review (Figure 1).

The relatively modest number of retrieved and included studies reflects the focused nature of the search strategy, which targeted the intersection of fNIRS methodology with clinically relevant language disorders or dyslexia. Although the search covered each database from inception, human functional NIRS has been used for brain functional mapping only since the early 1990s [11], and its application to clinically diagnosed language disorders and dyslexia remains a relatively small and emerging field.

### 2.4. Data Extraction

Data from the included studies were extracted and coded independently by two reviewers. To ensure the reliability of data extraction, all coding sheets were cross-checked independently. Discrepancies were resolved through discussion, and when consensus could not be reached, a third senior reviewer was consulted. All raters received standardized training before data extraction to ensure that they followed consistent criteria when performing their tasks.

Specifically, the following information was extracted from all included studies: first author, year, country, diagnostic group, fNIRS signal(s) analyzed, outcome metric type, region of interest (ROI), sample size (control/experimental), age (control/experimental).

To avoid effect-size dependency arising from multiple ROIs reported within the same study, only one ROI was selected for each outcome measure from each study. ROI selection followed a sequential, source-based procedure. First, when the original study explicitly designated a study-level primary or focal ROI, or a study-specific measurement region, that region was selected, provided that sufficient group-level data were available for the relevant task and outcome. Second, when no such study-level designation was provided, an outcome-specific ROI or channel was retained only when it was explicitly identified by the original authors in the abstract or main outcome-specific analysis and sufficient group-level data were available for extraction. Third, when the original study identified more than one comparably eligible ROI for the same outcome, the following theoretical hierarchy was applied: inferior frontal gyrus/inferior frontal cortex (IFG/IFC), followed by prefrontal cortex/dorsolateral prefrontal cortex/medial prefrontal cortex (PFC/DLPFC/medial PFC), and then temporoparietal junction/superior temporal gyrus (TPJ/STG). Eligible ROIs were not independently ranked according to effect-size magnitude, *p*-value, or effect direction; instead, original-study designations and outcome-specific reporting were followed as documented. The procedure was applied separately to each outcome measure; therefore, different ROIs could be selected for different hemoglobin measures within the same study. The selected ROI and the corresponding selection criterion for each study are summarized in Supplementary Table S2. When a study reported multiple independent samples, each sample was extracted separately and treated as an independent effect size.

### 2.5. Quality Assessment

Independent bias assessment was conducted by two reviewers using the JBI checklist for analytical cross-sectional studies [16]. Discrepancies between reviewers were resolved through discussion. For interpretive purposes, studies scoring 1–3 were classified as low quality, 4–6 as moderate quality, and 7–8 as high quality. No studies were excluded on the basis of quality score, in order to enhance transparency and ensure that all available evidence in this area was considered.

### 2.6. Statistical Analysis

Statistical analyses were conducted in R (v4.4.2; R Foundation for Statistical Computing, Vienna, Austria) using the meta and metafor packages. Included studies were grouped according to the reported fNIRS outcome metric (HbO, HbR, or β estimates). Studies were entered into quantitative synthesis when sufficient numerical data were available to derive an effect size for the experimental and control groups; otherwise, they were included in narrative synthesis only. The primary outcomes of interest were between-group differences in oxyhemoglobin (HbO), deoxyhemoglobin (HbR), and β estimates. For studies reporting group means and standard deviations, standardized mean differences (SMDs) and their 95% confidence intervals (CIs) were calculated for meta-analysis. Individual study results and pooled estimates were presented using forest plots, grouped by outcome type (HbO, HbR, and β). Where appropriate, publication bias was explored using funnel plots. Statistical heterogeneity was assessed using Cochran’s Q, I^2^, and τ^2^, with I^2^ indicating the proportion of total variability attributable to between-study heterogeneity and τ^2^ representing the absolute between-study variance in true effect sizes.

Separate random-effects meta-analyses were performed for HbO, HbR, and β outcomes. A random-effects model was prespecified because true effect sizes were expected to vary across studies owing to differences in ROIs, participant characteristics, and outcome definitions. Sensitivity analyses were conducted to examine the robustness of the pooled estimates. First, leave-one-out analyses were performed by sequentially omitting each study or independent effect estimate, as applicable. Additional sensitivity analyses were undertaken by excluding studies that used maximum-based metrics rather than mean-based measures, restricting analyses to pediatric and adult samples separately, and assessing the robustness of the pooled estimates to variation in specific frontal ROI definitions, so as to evaluate the influence of metric type, age group, and ROI selection on the overall results. Subgroup analyses were conducted according to disorder type to explore potential sources of heterogeneity and to determine whether diagnostic category influenced the magnitude or direction of the pooled effects.

Publication bias was initially evaluated using Egger’s regression test. Because the number of included studies was small (k < 10) and Egger’s test has inherently low statistical power in such scenarios, the non-parametric trim-and-fill method was additionally employed. This served as a sensitivity analysis to estimate the number of potentially missing studies and to assess their impact on the overall pooled effect size.

## 3. Results

The Results section is presented in relation to the four research questions stated in the Introduction. The pooled analyses of HbO, HbR, and β estimates address whether altered hemodynamic responses are present in individuals with language disorders or dyslexia relative to controls. Subgroup and sensitivity analyses further examine whether these effects vary according to ROI, age group, metric type, and disorder category. Comparisons across HbO, HbR, and β outcomes are used to assess the relative stability and comparability of different fNIRS-derived metrics, whereas disorder-type subgroup analyses are used to explore directional similarity or divergence across diagnostic categories.

### 3.1. Study Characteristics and Risk of Bias

A total of 10 studies comprising 11 independent samples were included in the review, including two studies on developmental language disorders, two studies on acquired language disorders (post-stroke aphasia), and six studies on dyslexia (Table 1). Fu et al. [17] reported separate child and adult samples, which are presented as two rows in Table 1 but represent a single publication. In terms of outcome definition, three studies reported model-derived β coefficients, whereas the remaining studies reported hemoglobin-based hemodynamic measures [17,18,19]. Most studies analyzed HbO and/or HbR signals, with a small number additionally reporting HbT or HbDiff [20,21]. Five studies were included in the HbO meta-analysis and 3 in the HbR meta-analysis [22,23,24,25,26]. Of the 10 publications, 8 provided sufficient data for quantitative synthesis, whereas 2 were included in the narrative review only [20,21]. The included studies involved both pediatric and adult populations, with some studies reporting independent age-defined cohorts that were extracted separately for analysis [17]. The primary ROIs were predominantly located in frontal regions, including the IFG, DLPFC, medial PFC, and broader PFC regions. All included studies met seven or eight of the eight JBI appraisal criteria (Table S3).

### 3.2. HbO Synthesis

**Figure 2:**
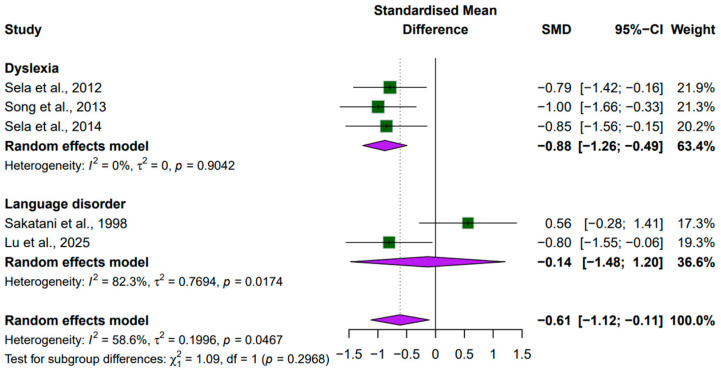
Forest plot showing the subgroup analysis of HbO outcomes by disorder category and the overall meta-analysis of HbO outcomes. Green squares represent individual-study effect estimates, and horizontal lines represent 95% confidence intervals. Purple diamonds represent pooled random-effects estimates. The solid vertical line at zero indicates no effect, whereas the dashed vertical line indicates the overall pooled effect estimate. The plotted studies were Sela et al. (2012) [25], Song et al. (2013) [24], Sela et al. (2014) [23], Sakatani et al. (1998) [26], and Lu et al. (2025) [22].

A total of five studies were included in the HbO meta-analysis, comprising 169 participants overall, with 81 in the experimental group and 88 in the control group. Of these studies, three focused on dyslexia and two on acquired language disorders. The regions of interest mainly involved frontal areas, including the PFC, DLPFC, and IFG. The random-effects model showed that HbO levels were significantly lower in the experimental group than in the control group (SMD = −0.61, 95% CI [−1.12, −0.11], *p* = 0.017). Moderate-to-substantial between-study heterogeneity was observed (Q = 9.65, *p* = 0.0467; I^2^ = 58.6%; τ^2^ = 0.1996; Figure 2). In addition, Elshazly et al. reported significantly higher HbDiff values in typically developing students than in those with dyslexia, suggesting greater oxygenation-related cortical activation in the control group [21]. Although HbDiff is not directly equivalent to HbO, this finding provides indirect narrative support for reduced oxygenation-related activation in the experimental group.

To preliminarily explore potential sources of heterogeneity, a subgroup analysis was conducted according to disorder type (Figure 2). In the dyslexia subgroup (k = 3), the pooled effect was statistically significant (SMD = −0.88, 95% CI [−1.26, −0.49]), with no apparent heterogeneity (Q = 0.20, *p* = 0.9042, I^2^ = 0.0%; τ^2^ = 0). In the acquired language disorder subgroup (k = 2), the pooled effect did not reach statistical significance (SMD = −0.14, 95% CI [−1.48, 1.20]), whereas substantial within-subgroup heterogeneity was observed (Q = 5.65, *p* = 0.0174; I^2^ = 82.3%; τ^2^ = 0.7694). However, the test for subgroup differences was not statistically significant (Q = 1.09, *p* = 0.2968), indicating that there is insufficient support for the notion that disorder category significantly explains between-group differences in HbO effect sizes. Given the small number of included studies, particularly in the language-disorder subgroup, these subgroup findings should be interpreted with caution.

**Figure 3:**
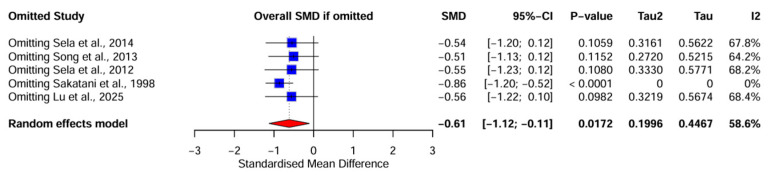
Leave-one-out sensitivity analysis of HbO outcomes. Blue squares represent the recalculated pooled SMDs after sequential omission of each study, and horizontal lines represent the corresponding 95% confidence intervals. The red diamond represents the original random-effects pooled estimate based on all included studies, with its width indicating the 95% confidence interval. The solid vertical line at SMD = 0 denotes no between-group difference, whereas the dashed vertical line marks the original overall pooled SMD. The omitted studies were Sela et al. (2014) [23], Song et al. (2013) [24], Sela et al. (2012) [25], Sakatani et al. (1998) [26], and Lu et al. (2025) [22].

To assess the robustness of the pooled estimate and further explore potential sources of heterogeneity, sensitivity analyses were performed. Subgroup difference tests according to metric type, ROI, and age group were all non-significant (*p* = 0.5186, 0.1897, and 0.1115, respectively), indicating that the available evidence did not support these variables as significant moderators of the pooled HbO effect. However, these findings should be interpreted with caution because several subgroups included only a small number of studies, particularly the children subgroup, which contained only one study. Leave-one-out analysis showed that the pooled HbO estimates remained consistently negative after omission of each study (SMD range: −0.86 to −0.51). However, statistical significance was retained only after omission of Sakatani et al. (1998) [26], whereas the confidence intervals crossed zero in the other four analyses. Heterogeneity ranged from 0% to 68.4% and decreased to 0% only after omission of Sakatani et al. (1998) [26]. These findings indicate directional consistency in the HbO effect, but limited robustness of its statistical significance (Figure 3).

### 3.3. HbR Synthesis

**Figure 4:**
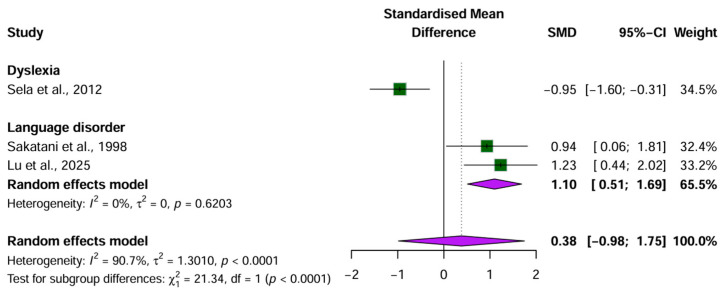
Forest plot showing the subgroup analysis of HbR outcomes by disorder category and the overall meta-analysis of HbR outcomes. Green squares represent individual-study effect estimates, and horizontal lines represent 95% confidence intervals. Purple diamonds represent pooled random-effects estimates. The solid vertical line at SMD = 0 denotes no between-group difference, whereas the dashed vertical line marks the overall pooled SMD. The plotted studies were Sela et al. (2012) [25], Sakatani et al. (1998) [26], and Lu et al. (2025) [22].

A total of three studies were included in the HbR meta-analysis, comprising 95 participants, with 44 in the experimental group and 51 in the control group. Of these, one study focused on dyslexia and two on acquired language disorders, with regions of interest mainly involving the PFC and medial PFC. The random-effects pooled point estimate was positive, but it was not statistically significant (SMD = 0.38, 95% CI [−0.98, 1.75], *p* = 0.5823). Substantial between-study heterogeneity was observed (Q = 21.58, *p* < 0.0001; I^2^ = 90.7%; τ^2^ = 1.3010; Figure 4).

To further investigate potential sources of heterogeneity, subgroup analyses were conducted by disorder type (Figure 4). In the dyslexia subgroup, only one study was available, yielding an effect size of SMD = −0.95, 95% CI [−1.60, −0.31], which suggested lower HbR levels in the experimental group than in the control group. Because this subgroup contained only a single study, within-subgroup heterogeneity could not be estimated, and no robust inference can be made for this disorder category. In the acquired language-disorder subgroup (k = 2), the pooled effect was statistically significant (SMD = 1.10, 95% CI [0.51, 1.69]), indicating higher HbR levels in the experimental group, with no evidence of heterogeneity (Q = 0.25, *p* = 0.6203; I^2^ = 0%; τ^2^ = 0). The test for subgroup differences was statistically significant (Q = 21.34, *p* < 0.0001), suggesting that disorder type may contribute to variation in HbR effect sizes. Nevertheless, given that the dyslexia subgroup was represented by only one study, these subgroup findings should be interpreted cautiously.

**Figure 5:**
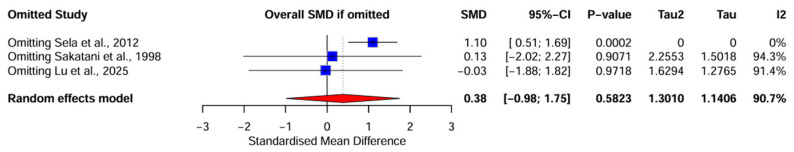
Leave-one-out sensitivity analysis of HbR outcomes. Blue squares represent the recalculated pooled SMDs after sequential omission of each study, and horizontal lines represent the corresponding 95% confidence intervals. The red diamond represents the original random-effects pooled estimate based on all included studies, with its width indicating the 95% confidence interval. The solid vertical line at SMD = 0 denotes no between-group difference, whereas the dashed vertical line marks the original overall pooled SMD. The omitted studies were Sela et al. (2012) [25], Sakatani et al. (1998) [26], and Lu et al. (2025) [22].

Sensitivity analyses were performed to assess the robustness of the pooled estimate and to explore additional sources of heterogeneity. Subgroup-difference tests based on metric type and ROI were both non-significant (both *p* = 0.5211), providing no clear evidence that either variable significantly moderated the pooled HbR effect. Sensitivity analysis by age group could not be conducted because all included studies were based on adult samples. Leave-one-out analysis further showed that the overall HbR findings were sensitive to individual studies. When Sela et al. (2012) [25] was excluded, the pooled effect became significantly positive (SMD = 1.10, 95% CI [0.51, 1.69], *p* = 0.0002), and heterogeneity dropped to 0%, suggesting that this study had a substantial influence on both the direction of the pooled effect and the observed heterogeneity. By contrast, omission of Sakatani et al. (1998) [26] or Lu et al. (2025) [22] yielded pooled effects close to zero that were no longer statistically significant, while heterogeneity remained high (I^2^ = 94.3% and 91.4%, respectively). This pattern suggests substantial inconsistency between Sela et al. (2012) [25] and the studies involving acquired language disorders. Taken together, these findings indicate that the overall HbR result was not robust and was disproportionately influenced by individual studies. Given the small number of included studies, as well as the limited data within several subgroups, these results should be interpreted with caution (Figure 5).

### 3.4. β Synthesis

**Figure 6:**
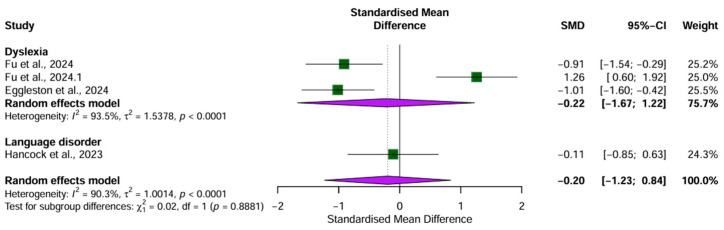
Forest plot showing the subgroup analysis of β outcomes by disorder category and the overall meta-analysis of β outcomes. Green squares represent individual effect estimates, and horizontal lines represent 95% confidence intervals. Purple diamonds represent pooled random-effects estimates. The solid vertical line at SMD = 0 denotes no between-group difference, whereas the dashed vertical line marks the overall pooled SMD. The plotted studies were Fu et al. (2024) [17], Eggleston et al. (2024) [18], and Hancock et al. (2023) [19]. Fu et al. (2024) [17] contributed separate child and adult samples, labelled “Fu et al., 2024” and “Fu et al., 2024.1”, respectively; “.1” does not indicate a separate publication.

Three studies contributed four independent samples/effect sizes, comprising 165 participants overall, with 79 in the experimental group and 86 in the control group. Three effect sizes for dyslexia were derived from two studies, whereas one effect size for developmental language disorder was derived from one study, and the reported regions of interest mainly involved the IFG, DLPFC, and medial PFC. The random-effects pooled estimate was negative but not statistically significant (SMD = −0.20, 95% CI [−1.23, 0.84], *p* = 0.7068). Substantial between-study heterogeneity was observed (Q = 30.89, *p* < 0.0001; I^2^ = 90.3%; τ^2^ = 1.0014; Figure 6). Notably, narrative evidence from Fu et al. (2016) [20] further suggests that developmental language disorder may also involve atypical hemodynamic patterns in language-related regions. In the group with specific language impairment, significant between-group differences were observed in oxygenated mean trends in the right inferior frontal cortex, with control trajectories remaining above those of the case group.

To further examine potential sources of heterogeneity, subgroup analyses were conducted according to disorder type (Figure 6). In the dyslexia subgroup, three independent effect estimates from two studies yielded a negative pooled point estimate (SMD = −0.22, 95% CI [−1.67, 1.22]). However, the confidence interval was wide and crossed zero, providing no clear evidence of a dyslexia-specific difference in β values. This subgroup also showed substantial heterogeneity (Q = 30.68, *p* < 0.0001; I^2^ = 93.5%; τ^2^ = 1.5378). In the developmental language-disorder subgroup, only one study was available, yielding an effect size of SMD = −0.11 (95% CI [−0.85, 0.63]). Although the direction of effect similarly suggested lower β values in the experimental group, the estimate was not statistically significant. Because this subgroup was represented by a single study, within-subgroup heterogeneity could not be estimated and the finding should be regarded as descriptive only. The test for subgroup differences was not statistically significant (Q = 0.02, *p* = 0.8881), indicating that there was no clear evidence that disorder type significantly moderated the pooled β effect.

**Figure 7:**
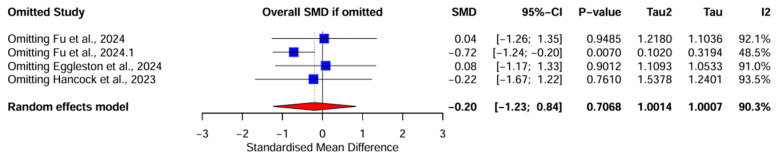
Leave-one-effect-out sensitivity analysis of β outcomes. Blue squares represent the recalculated pooled SMDs after sequential omission of each independent effect estimate, and horizontal lines represent the corresponding 95% confidence intervals. The red diamond represents the original random-effects pooled estimate based on all included studies, with its width indicating the 95% confidence interval. The solid vertical line marks SMD = 0, whereas the dashed vertical line marks the original overall pooled SMD. The omitted effect estimates were derived from Fu et al. (2024) [17], Eggleston et al. (2024) [18], and Hancock et al. (2023) [19]. Fu et al. (2024) [17] contributed separate child and adult samples, labelled “Fu et al., 2024” and “Fu et al., 2024.1”, respectively; “.1” does not indicate a separate publication.

Sensitivity analyses were performed to assess the robustness of the pooled estimate and to explore potential sources of heterogeneity. The ROI subgroup-difference test was not statistically significant (*p* = 0.1326), whereas the age-group difference was statistically significant (*p* < 0.0001). However, the age-related finding should be regarded as exploratory because the adult subgroup contained only one independent effect estimate, and age was confounded with study, task, and other sample characteristics. In the leave-one-effect-out analysis, pooled estimates ranged from −0.72 to 0.08. Omission of the adult sample from Fu et al. (2024) [17] yielded a statistically significant negative effect (SMD = −0.72, 95% CI [−1.24, −0.20], *p* = 0.0070) and reduced heterogeneity to 48.5%, whereas the other estimates remained non-significant and highly heterogeneous. These findings indicate that the β synthesis was sensitive to the composition of the small evidence base (Figure 7).

### 3.5. Publication Bias

Egger’s test showed no statistically significant evidence of funnel plot asymmetry for the HbO (*p* = 0.0893), HbR (*p* = 0.2798), or β analyses (*p* = 0.5035). Trim-and-fill analysis did not impute any additional effects. However, because each analysis contained only a small number of studies, these procedures had very limited statistical power. In particular, publication bias and small-study effects cannot be ruled out for the HbO analysis. These findings only indicate that funnel-plot asymmetry was not detected, not that publication bias was absent.

## 4. Discussion

### 4.1. Summary of Evidence

The present synthesis showed that different hemoglobin parameters provided varying levels of evidence across fNIRS studies of language disorders and dyslexia. The pooled HbO effect was significantly negative and remained directionally consistent in leave-one-out analyses, although its statistical significance was not consistently retained. HbR and β findings were non-significant, highly heterogeneous, and more sensitive to individual studies or samples. Therefore, HbO, HbR, and β should not be regarded as equivalent indicators, because they may differ in their sensitivity, stability, and cross-study comparability in detecting between-group differences. fNIRS reproducibility has been shown to vary with data quality, response modelling, and analysis-pipeline choices [27], which may partly explain inconsistent findings across studies using different outcome measures or analytical methods [28]. Previous reviews have also shown that fNIRS studies frequently prioritize HbO and report chromophores inconsistently [29].

These findings should be interpreted within the exploratory transdiagnostic framework of this review. Although dyslexia, developmental language disorder, and post-stroke aphasia differ in onset, etiology, and neurobiological basis, they all involve language- or reading-related processing and have been investigated using fNIRS measures. The rationale for the synthesis was therefore to examine whether hemodynamic response patterns converge or diverge across disorders within this shared functional domain, rather than to assume that the included conditions are etiologically homologous. Accordingly, the aggregate effects should be interpreted as preliminary evidence of cross-diagnostic convergence or divergence at the level of task-evoked hemodynamic responses, not as evidence of a shared disease continuum or common underlying mechanism.

In the HbO subgroup analysis, dyslexia showed a clearer negative pooled effect, whereas the estimate for acquired language disorders was close to zero and highly uncertain. The non-significant subgroup-difference test should not be interpreted as evidence that the two diagnostic categories showed equivalent response patterns, given the small number of studies.

Among the studies synthesized, the reported ROIs were concentrated mainly in frontal regions, particularly the IFG and broader prefrontal cortex, with only a small number of studies involving temporoparietal regions such as the TPJ. Given the established role of frontal language-related regions, especially the IFG, in phonological processing, syntactic processing, and language control, this finding may reflect lower processing efficiency or less effective engagement of frontal language networks in individuals with language disorders and dyslexia. This interpretation is broadly consistent with previous neuroimaging evidence. For example, Li et al. reported hypoactivation of the left inferior frontal cortex in children with dyslexia across different writing systems [30]. However, because the present study did not systematically compare all cortical regions and the available evidence was weighted more heavily toward frontal ROIs, the current findings are better interpreted as supporting the involvement of IFG-related frontal regions as relatively consistently affected areas, rather than identifying the IFG as the sole or definitive core region. These fNIRS findings are broadly consistent with, but more spatially constrained than, evidence from fMRI studies. In dyslexia, fMRI meta-analyses and reviews have reported atypical activation within the left-hemisphere reading network, including inferior frontal, temporoparietal, and occipitotemporal regions [31,32]. The reduced HbO pattern observed in the present synthesis is therefore generally compatible with evidence of less efficient recruitment of language- and reading-related cortical systems. Accordingly, convergence with the fMRI literature primarily concerns its frontal component rather than the whole language or reading network. For developmental language disorder and post-stroke aphasia, MRI/fMRI studies also suggest atypical language-network organization and functional reorganization, including altered activation/connectivity patterns in developmental conditions and compensatory recruitment of preserved left-hemisphere or right-hemisphere regions after stroke [33,34,35]. Therefore, the present fNIRS findings should be interpreted as complementary to, rather than directly equivalent to, the broader fMRI evidence.

Compared to HbO, the HbR results were substantially less consistent. Although the pooled HbR effect was positive in direction, it did not reach statistical significance, and the substantial between-study heterogeneity indicates that current evidence is insufficient to support a stable and uniform pattern of HbR alteration across acquired language disorders and dyslexia. On one hand, this may be related to the limited number of studies included in HbR analysis, with only 3 articles incorporated in this study, resulting in limited statistical power. On the other hand, fNIRS signals contain multiple physiological and stimulus-dependent components whose relative contributions may vary across experimental settings [36]. Such variability may further complicate comparison of HbR findings across a small and methodologically heterogeneous literature.

The results for β coefficients were also inconclusive. The pooled β effect was negative in direction but did not reach statistical significance. Unlike HbO and HbR, β estimates are model-derived parameters that may depend on analytical choices such as model specification and preprocessing strategies, resulting in limited cross-study comparability [37]. Therefore, although β values may capture meaningful task-related activation, the cross-study comparability may be inferior to directly reported hemodynamic metrics. This may partly explain why the β analysis did not yield a clear or stable pooled effect in the present study.

Subgroup findings varied across outcomes. Disorder category did not significantly explain the HbO or β effects, whereas the HbR subgroup difference was statistically significant. However, the HbR dyslexia subgroup and the β developmental language-disorder subgroup were each represented by only one study, and the ROI- and metric-type comparisons were also based on sparse data. These findings should therefore be regarded as exploratory. Age-related variability remains important because the included samples ranged from school-aged children to older adults with post-stroke aphasia. Cortical maturation, language-network organization, and neurovascular coupling may influence fNIRS-derived responses across the lifespan. Although a significant age-group difference was observed for β, the adult category was represented only by the adult sample from Fu et al. (2024) [17], and age was confounded with study, task, and disorder category. This result therefore does not establish age as an independent moderator. Leave-one-out analyses also showed different levels of robustness across outcomes. The HbO estimates remained negative, but statistical significance was not consistently retained. Omission of Sakatani et al. (1998) [26] eliminated heterogeneity, indicating that this study contributed substantially to the observed variability. The HbR findings were highly sensitive to individual studies. The β result was particularly influenced by the adult sample from Fu et al. (2024) [17], whose omission produced a statistically significant negative pooled effect and reduced heterogeneity.

### 4.2. Limitations

No previous meta-analysis appears to have synthesized fNIRS findings in language disorders and dyslexia using a comprehensive search strategy; however, several limitations should be considered when interpreting the results. First, the number of included studies was limited and unevenly distributed across outcome measures, with only five studies contributing to the HbO analysis, three to the HbR analysis, and three studies contributing four effect estimates to the β analysis. This small evidence base may reduce the precision of pooled estimates and the stability of heterogeneity, subgroup, and sensitivity analyses. Therefore, the significant HbO finding should be regarded as a preliminary signal requiring further confirmation, and the non-significant HbR and β findings should be interpreted cautiously rather than as evidence of no effect. Given the limited number of studies and unevenly distributed study characteristics, meta-regression was not performed. In addition, the wide and uneven age distribution across studies may have contributed to between-study heterogeneity, but the limited number of studies prevented a robust evaluation of age as an independent moderator. Second, publication-bias assessments were severely underpowered because each meta-analysis included only a small number of studies. Consequently, the non-significant Egger’s tests and the absence of trim-and-fill imputations should not be interpreted as evidence against publication bias. In particular, given that the HbO analysis included only five studies, the possibility of publication bias cannot be excluded. Third, to avoid effect-size dependency, only one ROI was selected for each outcome measure from each study for the main analysis. Although this approach improved cross-study comparability and reduced statistical dependence, it may also have limited the extent to which within-study variability was represented. Moreover, because the selected ROIs were mainly concentrated in frontal and prefrontal language-related regions, the pooled findings should be interpreted as reflecting the available evidence from these commonly reported ROIs rather than whole-brain hemodynamic alterations during language or reading tasks. In addition, the inclusion of both developmental and acquired conditions introduced substantial clinical and etiological heterogeneity. Although disorder-stratified results were reported where data permitted, several diagnostic subgroups were represented by only one study, limiting robust comparisons across diagnostic categories. The pooled findings should therefore be interpreted cautiously.

### 4.3. Future Directions

Future research should include larger samples, more balanced representation across disorder categories, and more standardized ROI selection and outcome reporting to improve cross-study comparability and further clarify both shared and disorder-specific features of language disorders and dyslexia.

## 5. Conclusions

This study systematically synthesized fNIRS evidence related to language disorders and dyslexia. The current findings indicate that, among the studies included, HbO was the only outcome to show a statistically significant overall between-group difference and appeared to provide relatively more consistent evidence across studies. In contrast, findings based on HbR and β were less stable and showed weaker cross-study consistency. However, because the available evidence was limited, clinically heterogeneous, and unevenly distributed across outcome measures, these findings should be interpreted as preliminary and hypothesis-generating rather than confirmatory. The observed HbO pattern should therefore be regarded as an exploratory cross-diagnostic signal within the available literature, rather than as evidence of a shared disease continuum or common underlying mechanism.

## Figures and Tables

**Table 1:** Characteristics of included studies.

First Author	Year	Country	Diagnostic Group	fNIRS Signal Analyzed	Outcome Metric Type	Region of Interest (ROI)	Sample Size (Control Group)	Sample Size (Experimental Group)	Age (Years) (Control)	Age (Years) (Experimental)
Lu et al. [22]	2025	China	Language disorder (Poststroke Aphasia)	HbO, HbR	mean activation	L IFG; medial PFC	15	15	52 ± 17	54 ± 13
Elshazly et al. [21]	2024	United Arab Emirates	Dyslexia	HbDiff	hemoglobin difference	Frontal cortex	7	7	NR (third-grade students)	NR (third-grade students)
Fu et al. [17] (child sample)	2024	China	Dyslexia	HbO	β coefficient	R IFG	24	20	10.6 ± 0.6	11 ± 0.7
Fu et al. [17] (adult sample)	2024	China	Dyslexia	HbO	β coefficient	R IFG	23	20	19.7 ± 1.5	19.6 ± 1.0
Eggleston et al. [18]	2024	USA	Dyslexia	HbO	β coefficient	L vIFG	25	25	7.46 ±1.33	9.77 ± 1.19
Hancock et al. [19]	2023	USA	Language disorder (developmental language disorder)	HbO	β coefficient	L DLPFC	14	14	11.4 ± 1.75	11.54 ± 2.03
Fu et al. [20]	2016	USA	Language disorder (specific language impairment—SLI)	HbO, HbR, HbT	FDA mean trend	R IFC; L TPJ	15	15	8–12	8–12
Sela et al. [23]	2014	USA	Dyslexia	HbO	mean activation	L PFC	17	17	25.06 ± 2.384	25.65 ± 2.668
Song et al. [24]	2013	China	Dyslexia	HbO	mean activation	L DLPFC	20	20	10.37 ± 0.42	10.12 ± 0.81
Sela et al. [25]	2012	USA	Dyslexia	HbO, HbR	peak/maximum change	L PFC	23	19	25.05 ± 2.10	25.36 ± 3.23
Sakatani et al. [26]	1998	China	Language disorder (Poststroke Aphasia)	HbO, HbR	peak/maximum change	L PFC	13	10	50.7 ± 7.9	56.9 ± 7

Note. HbO, oxygenated hemoglobin; HbR, deoxygenated hemoglobin; HbT, total hemoglobin; FDA, functional data analysis; ROI, region of interest; IFG, inferior frontal gyrus; vIFG, ventral inferior frontal gyrus; DLPFC, dorsolateral prefrontal cortex; PFC, prefrontal cortex; TPJ, temporoparietal junction; SLI, specific language impairment; NR, not reported. Age is reported as mean ± SD or range. The child and adult samples reported by Fu et al. [17] are presented separately but originate from the same publication.

## Data Availability

The original contributions presented in this study are included in the article and Supplementary Materials. Further inquiries can be directed to the corresponding author.

## Supplementary Materials

The following supporting information can be downloaded at: https://www.mdpi.com/article/10.3390/diagnostics16152388/s1, Table S1: PubMed, Embase, and Web of Science search strategies. Table S2: Study-level documentation of the reported ROIs, ROI selected for each outcome, and corresponding selection criterion. Table S3: The results of the risk of bias assessment for each included article.

## References

[1] Bishop D.V.M., Snowling M.J., Thompson P.A., Greenhalgh T., CATALISE-2 consortium (2017). Phase 2 of CATALISE: A Multinational and Multidisciplinary Delphi Consensus Study of Problems with Language Development: Terminology. J. Child Psychol. Psychiatry.

[2] Krishnan S., Watkins K.E., Bishop D.V.M. (2016). Neurobiological Basis of Language Learning Difficulties. Trends Cogn. Sci..

[3] Kiran S. (2012). What Is the Nature of Poststroke Language Recovery and Reorganization?. ISRN Neurol..

[4] Peterson R.L., Pennington B.F. (2012). Developmental Dyslexia. Lancet.

[5] Lyon G.R., Shaywitz S.E., Shaywitz B.A. (2003). A Definition of Dyslexia. Ann. Dyslexia.

[6] Norton E.S., Beach S.D., Gabrieli J.D. (2015). Neurobiology of Dyslexia. Curr. Opin. Neurobiol..

[7] Butler L.K., Kiran S., Tager-Flusberg H. (2020). Functional Near-Infrared Spectroscopy in the Study of Speech and Language Impairment across the Life Span: A Systematic Review. Am. J. Speech-Lang. Pathol..

[8] Michel C.M., Brunet D. (2019). EEG Source Imaging: A Practical Review of the Analysis Steps. Front. Neurol..

[9] De Vos M., Riès S., Vanderperren K., Vanrumste B., Alario F.-X., Van Huffel S., Burle B. (2010). Removal of Muscle Artifacts from EEG Recordings of Spoken Language Production. Neuroinform.

[10] Huppert T.J., Diamond S.G., Franceschini M.A., Boas D.A. (2009). HomER: A Review of Time-Series Analysis Methods for near-Infrared Spectroscopy of the Brain. Appl. Opt..

[11] Ferrari M., Quaresima V. (2012). A Brief Review on the History of Human Functional Near-Infrared Spectroscopy (fNIRS) Development and Fields of Application. NeuroImage.

[12] Quaresima V., Bisconti S., Ferrari M. (2012). A Brief Review on the Use of Functional Near-Infrared Spectroscopy (fNIRS) for Language Imaging Studies in Human Newborns and Adults. Brain Lang..

[13] Rossi S., Telkemeyer S., Wartenburger I., Obrig H. (2012). Shedding Light on Words and Sentences: Near-Infrared Spectroscopy in Language Research. Brain Lang..

[14] Girolamo T., Butler L., Canale R., Aslin R.N., Eigsti I.-M. (2024). fNIRS Studies of Individuals with Speech and Language Impairment Underreport Sociodemographics: A Systematic Review. Neuropsychol. Rev..

[15] Page M.J., McKenzie J.E., Bossuyt P.M., Boutron I., Hoffmann T.C., Mulrow C.D., Shamseer L., Tetzlaff J.M., Akl E.A., Brennan S.E. (2021). The PRISMA 2020 Statement: An Updated Guideline for Reporting Systematic Reviews. BMJ.

[16] Joanna Briggs Institute (2020). Checklist for Analytical Cross Sectional Studies.

[17] Fu Y., Yan X., Mao J., Su H., Cao F. (2024). Abnormal Brain Activation during Speech Perception and Production in Children and Adults with Reading Difficulty. npj Sci. Learn..

[18] Eggleston R.L., Marks R.A., Sun X., Yu C.-L., Zhang K., Nickerson N., Hu X., Caruso V., Kovelman I. (2024). Lexical Morphology as a Source of Risk and Resilience for Learning to Read with Dyslexia: An fNIRS Investigation. J. Speech Lang. Hear. Res..

[19] Hancock A.S., Warren C.M., Barrett T.S., Bolton D.A.E., Gillam R.B. (2023). Functional Near-infrared Spectroscopy Measures of Neural Activity in Children with and without Developmental Language Disorder during a Working Memory Task. Brain Behav..

[20] Fu G., Wan N.J.A., Baker J.M., Montgomery J.W., Evans J.L., Gillam R.B. (2016). A Proof of Concept Study of Function-Based Statistical Analysis of fNIRS Data: Syntax Comprehension in Children with Specific Language Impairment Compared to Typically-Developing Controls. Front. Behav. Neurosci..

[21] Elshazly E.M.E., Mostafa H., Safi M.F. (2024). Correlation between the Cortical Activation Studied by Functional near Infrared Spectroscopy Neuroimaging (fNIRS) with Performance of 3rd Grade Students. Int. J. Technol.-Enhanc. Educ..

[22] Lu C., Wang M., Zhan L., Lu M. (2025). Unveiling Cognitive Interference: fNIRS Insights into Poststroke Aphasia during Stroop Tasks. Neural Plast..

[23] Sela I., Izzetoglu M., Izzetoglu K., Onaral B. (2014). A Functional Near-Infrared Spectroscopy Study of Lexical Decision Task Supports the Dual Route Model and the Phonological Deficit Theory of Dyslexia. J. Learn. Disabil..

[24] Song R., Zhang J., Wang B., Zhang H., Wu H. (2013). A Near-Infrared Brain Function Study of Chinese Dyslexic Children. Neurocase.

[25] Sela I., Izzetoglu M., Izzetoglu K., Onaral B. (2012). A Working Memory Deficit among Dyslexic Readers with No Phonological Impairment as Measured Using the N-Back Task: An fNIR Study. PLoS ONE.

[26] Sakatani K., Xie Y., Lichty W., Li S., Zuo H. (1998). Language-Activated Cerebral Blood Oxygenation and Hemodynamic Changes of the Left Prefrontal Cortex in Poststroke Aphasic Patients: A near-Infrared Spectroscopy Study. Stroke.

[27] Yücel M.A., Luke R., Mesquita R.C., von Lühmann A., Mehler D.M.A., Lührs M., Gemignani J., Abdalmalak A., Albrecht F., de Almeida Ivo I. (2025). fNIRS Reproducibility Varies with Data Quality, Analysis Pipelines, and Researcher Experience. Commun. Biol..

[28] Yücel M.A., Lühmann A.V., Scholkmann F., Gervain J., Dan I., Ayaz H., Boas D., Cooper R.J., Culver J., Elwell C.E. (2021). Best Practices for fNIRS Publications. Neurophotonics.

[29] Kinder K.T., Heim H.L.R., Parker J., Lowery K., McCraw A., Eddings R.N., Defenderfer J., Sullivan J., Buss A.T. (2022). Systematic Review of fNIRS Studies Reveals Inconsistent Chromophore Data Reporting Practices. Neurophotonics.

[30] Li Y., Bi H.-Y. (2022). Comparative Research on Neural Dysfunction in Children with Dyslexia under Different Writing Systems: A Meta-Analysis Study. Neurosci. Biobehav. Rev..

[31] Richlan F., Kronbichler M., Wimmer H. (2009). Functional Abnormalities in the Dyslexic Brain: A Quantitative Meta-Analysis of Neuroimaging Studies. Hum. Brain Mapp..

[32] Martin A., Kronbichler M., Richlan F. (2016). Dyslexic Brain Activation Abnormalities in Deep and Shallow Orthographies: A Meta-Analysis of 28 Functional Neuroimaging Studies. Hum. Brain Mapp..

[33] Riès S.K., Dronkers N.F., Knight R.T. (2016). Choosing Words: Left Hemisphere, Right Hemisphere, or Both? Perspective on the Lateralization of Word Retrieval: Cerebral Lateralization of Word Retrieval. Ann. N. Y. Acad. Sci..

[34] Tilton-Bolowsky V., Stockbridge M.D., Hillis A.E. (2024). Remapping and Reconnecting the Language Network after Stroke. Brain Sci..

[35] Liégeois F., Mayes A., Morgan A. (2014). Neural Correlates of Developmental Speech and Language Disorders: Evidence from Neuroimaging. Curr. Dev. Disord. Rep..

[36] Reddy P., Izzetoglu M., Shewokis P.A., Sangobowale M., Diaz-Arrastia R., Izzetoglu K. (2021). Evaluation of fNIRS Signal Components Elicited by Cognitive and Hypercapnic Stimuli. Sci. Rep..

[37] Hou X., Zhang Z., Zhao C., Duan L., Gong Y., Li Z., Zhu C. (2021). NIRS-KIT: A MATLAB Toolbox for Both Resting-State and Task fNIRS Data Analysis. Neurophotonics.

