# Supplementary material for: fNIRS Hemodynamic Alterations in Developmental and Acquired Language Disorders and Dyslexia: An Exploratory Transdiagnostic Systematic Review and Meta-Analysis
Source: Diagnostics (Basel). 2026 Jul 29;16(15):2388. doi: 10.3390/diagnostics16152388 (PMC13465496; doi:10.3390/diagnostics16152388)
Supplement: Supplementary file 1 [file diagnostics-16-02388-s001.zip › diagnostics-4293272-supplementary/Supplementary Files/Revised Table S1.pdf]

Table S1: PubMed, Embase, and Web of Science search strategies

Database	Standardized final search strategy
PubMed	(("Near-Infrared Spectroscopy"[Mesh] OR "functional near infrared spectroscopy"[tiab] OR "functional near-infrared spectroscopy"[tiab] OR "near infrared spectroscopy"[tiab] OR "near-infrared spectroscopy"[tiab] OR "near infrared spectrometry"[tiab] OR "near-infrared spectrometry"[tiab] OR fnirs[tiab] OR nirs[tiab]) AND ("Language Disorders"[Mesh] OR "Aphasia"[Mesh] OR "Dyslexia"[Mesh] OR language disorder*[tiab] OR acquired language disorder*[tiab] OR developmental language disorder*[tiab] OR language disabilit*[tiab] OR language impairment*[tiab] OR "specific language impairment"[tiab] OR aphasia*[tiab] OR dyslexi*[tiab] OR alexia*[tiab] OR reading disorder*[tiab] OR developmental reading disorder*[tiab] OR "developmental dyslexia"[tiab] OR reading disabilit*[tiab]))
Embase	(('near infrared spectroscopy'/exp OR 'functional near infrared spectroscopy':ti,ab,kw OR 'functional near-infrared spectroscopy':ti,ab,kw OR 'near infrared spectroscopy':ti,ab,kw OR 'near-infrared spectroscopy':ti,ab,kw OR 'near infrared spectrometry':ti,ab,kw OR 'near-infrared spectrometry':ti,ab,kw OR fnirs:ti,ab,kw OR nirs:ti,ab,kw) AND ('language disorder'/exp OR aphasia/exp OR dyslexia/exp OR 'language disorder*':ti,ab,kw OR 'acquired language disorder*':ti,ab,kw OR 'developmental language disorder*':ti,ab,kw OR 'language disabilit*':ti,ab,kw OR 'language impairment*':ti,ab,kw OR 'specific language impairment':ti,ab,kw OR aphasia*:ti,ab,kw OR dyslexi*:ti,ab,kw OR alexia*:ti,ab,kw OR 'reading disorder*':ti,ab,kw OR 'developmental reading disorder*':ti,ab,kw OR 'developmental dyslexia':ti,ab,kw OR 'reading disabilit*':ti,ab,kw))
Web of Science	TS=(("functional near infrared spectroscopy" OR "functional near-infrared spectroscopy" OR "near infrared spectroscopy" OR "near-infrared spectroscopy" OR "near infrared spectrometry" OR "near-infrared spectrometry" OR fNIRS OR NIRS) AND ("language disorder*" OR "acquired language disorder*" OR "developmental language disorder*" OR "language disabilit*" OR "language impairment*" OR "specific language impairment" OR aphasia* OR dyslexi* OR alexia* OR "reading disorder*" OR "developmental reading disorder*" OR "developmental dyslexia" OR "reading disabilit*"))
