# Supplementary material for: fNIRS Hemodynamic Alterations in Developmental and Acquired Language Disorders and Dyslexia: An Exploratory Transdiagnostic Systematic Review and Meta-Analysis
Source: Diagnostics (Basel). 2026 Jul 29;16(15):2388. doi: 10.3390/diagnostics16152388 (PMC13465496; doi:10.3390/diagnostics16152388)
Supplement: Supplementary file 1 [file diagnostics-16-02388-s001.zip › diagnostics-4293272-supplementary/Supplementary Files/Revised Table S3.pdf]

Table S3. The results of the risk of bias assessment for each included article.

Study	Q1	Q2	Q3	Q4	Q5	Q6	Q7	Q8	Total
Lu et al. 2025	1	1	1	1	1	1	1	1	8
Elshazly et al. 2024	1	1	1	1	1	1	0	1	7
Fu et al. 2024	1	1	1	1	1	1	1	1	8
Eggleston et al. 2024	1	1	1	1	1	1	1	1	8
Hancock et al. 2023	1	1	1	1	1	1	1	1	8
Fu et al. 2016	1	1	1	1	1	1	1	1	8
Sela et al. 2014	1	1	1	1	1	1	1	1	8
Song et al. 2013	1	1	1	1	1	1	1	1	8
Sela et al. 2012	1	1	1	1	1	1	1	1	8
Sakatani et al. 1998	1	1	1	1	1	1	1	1	8

Note: Q1–Q8 correspond to the eight items of the JBI Critical Appraisal Checklist for Analytical Cross-Sectional Studies; 1 = yes, 0 = no.

Q1. Were the criteria for inclusion in the sample clearly defined?

Q2. Were the study subjects and the setting described in detail?

Q3. Was the exposure measured in a valid and reliable way?

Q4. Were objective, standard criteria used for measurement of the condition?

Q5. Were confounding factors identified?

Q6. Were strategies to deal with confounding factors stated?

Q7. Were the outcomes measured in a valid and reliable way?

Q8. Was appropriate statistical analysis used?
